# Moderating effect of health knowledge and mental health on the association between undergraduates’ attitudes toward help-seeking and internet addiction

**DOI:** 10.1186/s12889-025-23538-x

**Published:** 2025-07-02

**Authors:** Mensah Prince Osiesi, Sunday Ade Adeniran, Oluwayemisi Damilola Akomolafe

**Affiliations:** 1 Department of Guidance and Counseling, Faculty of Education, Federal University Oye-Ekiti, Ekiti State, Oye, Nigeria; 2 Department of Science Education, Faculty of Education, Federal University Oye-Ekiti, Ekiti State, Oye, Nigeria; 3 Department of Guidance and Counseling Education, Faculty of Education, Federal University Oye-Ekiti, Ekiti State, Oye, Nigeria

**Keywords:** Attitudes toward help-seeking, Internet addiction, Health knowledge, Mental health, Moderating effect

## Abstract

This study examined the moderating effect of health knowledge and mental health on the association between undergraduates’ attitudes toward help-seeking and internet addiction in three public universities in southwest Nigeria. Underpinned by the Uses and Gratification and the Functional-Interactive-Critical Health Literacy Theories, the study utilised the correlational research design. This study’s population comprised all undergraduates from universities in southwest Nigeria, with 1,684 participants. Data were gathered using the Internet Addiction Questionnaire (α = 0.82), Attitude to Help-Seeking Questionnaire (α = 0.84), Mental Health Questionnaire (α = 0.91), and the Health Knowledge Questionnaire (α = 0.94). The data were analysed using SPSS version 26.0's Hayes macro procedure. The characteristics of the respondents were summarised using descriptive statistics, and hypotheses were tested at a 0.05% significance level using inferential statistics (Hayes macro process V4.0, Model 2). Findings reveal that undergraduates’ attitudes toward help-seeking had impact on their addiction to the internet, health knowledge had no impact undergraduates’ internet addiction, mental health had no impact internet addiction among undergraduates, and health knowledge and mental health of undergraduates had no moderating effect on the relationship between attitudes toward help-seeking and internet addiction. The study recommends that further awareness campaigns on the essence of having positive attitudes toward help-seeking and the adverse effects of internet addiction should be organised in Nigerian public universities. Implications for theory, practice, and policy were discussed.

**Supplementary Information:**

The online version contains supplementary material available at 10.1186/s12889-025-23538-x.

## Introduction

The internet is supposed to facilitate social interaction, relaxation, and information searching; however, an over-reliance on it could result in addiction [5]. Since the internet is so accessible, students (especially university students) rely on it extensively for their academic and non-academic endeavours [3, 16]. Research affirms that too much internet use hurts mental health, which can result in poor social connections, depression, and other unfavourable outcomes [2]. Education specialists, psychologists, and social pathologists have reiterated the possible harm caused by excessive internet use and the associated psychological and physical adverse effects [7, 78]. According to Kuss et al. [37], individuals who spend more than 38 h a week online and lose control over their behaviour are said to have an internet addiction. Zewude et al. [78] assert that internet addiction is typically defined as an impulse control disorder that is very similar to pathological gambling and does not include the use of intoxicating drugs.

The health knowledge of the populace (undergraduates in this case) is essential to achieving the third Sustainable Development Goal (SDG), which is ensuring and promoting healthy lives for all at all ages. This knowledge can best be acquired through education [66]. Strong health knowledge can support students' mental health, and is crucial to their academic performance, engagement, or attitudes toward learning [10]. Because of health knowledge, students' attitudes toward help-seeking lessen the threat to their mental health when it is threatened. University students regularly face health-related and non-health-related challenges [28]. When students' mental health is in danger, their attitudes toward help-seeking, which comes from their health knowledge, will lessen that danger [10].

People's attitudes toward help-seeking are complicated and differ widely from one another [68], and they are positively impacted by their health literacy and knowledge [25]. Attitudes can affect individuals' preferences for help-seeking, and among young people (including students), a negative attitude towards help-seeking is a leading cause of health challenges [10, 57]. Three factors influence one's attitude towards professional and psychological aid: choosing to handle problems alone, being receptive to receiving help, and appreciating receiving support [68].

An individual's mental health includes social, emotional, and psychological well-being. Every stage of life, from childhood and adolescence to maturity, is impacted by mental health, which is defined as an individual's way of thinking, feeling, and acting, particularly regarding stress management, social interactions, and making wise decisions [15]. The World Health Organisation [73] defines mental health as a state of well-being in which individuals are conscious of their ability to cope with daily stressors, work productively, and give back to the community.

Mental health is an essential human right for achieving economic, societal, and personal development [26]. It affects people's ability to communicate, make a living, engage, think, and enjoy everyday activities [39, 66]. According to Balogun et al. [11] and Fadiya and Akinola [23], students' mental health has not received the required attention, especially in the Nigerian education space. According to studies, students' mental health is crucial for maintaining their physical health, improving their academic performance, fostering better social relationships, and realising their long-term goals [39, 48]. Students with low health knowledge may become lonely, isolated, sad, and suicidal because of their lack of awareness of local health options, which could result in a poor attitude toward seeking help [38] and become addicted to the internet.

Therefore, this study examines the moderating effect of health knowledge and mental health on the association between undergraduates’ attitudes toward help-seeking and internet addiction in selected public universities in southwest Nigeria. This study is important for understanding how these factors influence mental health and help-seeking behaviours, particularly among undergraduates (a group known to be vulnerable to both issues). This research can provide valuable insights into developing targeted interventions and support systems for students, ultimately improving their mental well-being, academic success, and healthy internet use.

### Problem statement

Internet addiction presents several significant dangers that affect both physical and mental health, as well as social and occupational functioning. These dangers include but not limited to mental health problems, sleep disorders, physical health problems, social isolation and relationship strain, cognitive and occupational impairment, behavioural and emotional symptoms, increased exposure to cyberbullying, online harassment, risky behaviours such as compulsive gambling or shopping, and substance abuse [7]. Particularly, university students are known to experience mental health issues, specifically anxiety, depression, and suicide [6, 31].

Despite the possibility that these students' poor health knowledge and internet addiction have impacted their mental health, several studies confirm their poor attitude or reluctance toward seeking help. This has been attributed to barriers like stigma, a shortage of qualified professionals, and financial concerns [60]. Although prior research has mainly evaluated the relationships between students' mental health, health knowledge, and help-seeking behaviour, little is known about how these relate to students' internet addiction or how mental health and health knowledge may moderate the relationship between students' internet addiction and attitudes toward seeking help. The current study, therefore, investigates the moderating role of mental health and health knowledge on the relationship between undergraduates' attitudes toward help-seeking and internet addiction in three public universities in southwest Nigeria.

## Literature review

### Impact of attitude towards help-seeking on undergraduates’ internet addiction

The internet has become essential to university students' lives because of its extensive use [61]. In addition to providing students with extensive exploration spaces and enabling online learning and social media interactions [18], the internet's increasing dependence also raises concerns about internet addiction and its detrimental effects on students' mental health [14, 21]. One way to describe help-seeking is as an adaptive coping strategy involving seeking external help for mental health issues [5]. People frequently put off or postpone getting treatment for mental health issues, even though it is essential for getting the right therapy and enhancing mental wellness [76]. Help-seeking behaviour is essential for better mental health, and students' unwillingness to actively seek professional help has also been linked to a general mistrust of mental health services [52, 76]). The attitude of undergraduates toward getting help for mental health concerns at the University of Ilorin, Nigeria, was examined by Muhammad et al. [52]. The research indicates that most students prefer to discuss their mental health issues with friends and family rather than pursuing psychotherapy or medical assistance. Lu et al. [45] examined the relationship between social support and internet addiction among Chinese college freshmen. Findings indicate that internet addiction harms students’ social support.

### Impact of health knowledge on undergraduates’ Internet addiction

Health knowledge includes a precise and in-depth understanding of the causes, symptoms, transmission, treatment, resources, and services related to health (assessed as perceived knowledge in this study) [10]. Students' health knowledge can prevent the spread of illnesses and guarantee the advancement of both individual and community health [32]. Accurate information about illnesses and general health concerns contributes to debunking myths about pandemics and endemics, raising awareness of the risk of infection, and improving self-defense against health traumas [58]. Research has indicated that an individual's attitudes toward health-related issues, such as help-seeking, are predicted by their level of health knowledge [62, 79].

As technology develops, web technology, cyberspace, and technological integration have become commonplace in educational institutions. Social media and the internet have become essential components of human civilisation. Students' internet use has been crucial to their learning habits [33]. Langarizadeh et al. [40] investigated how information literacy predicts internet addiction among medical sciences students at Iran University. Results indicate a strong negative correlation between internet addiction and information literacy,the more information literate a person is, the less addicted they are to the Internet, and as a result, society's overall health will improve. Maheri et al. [46] investigated how Tehran University medical sciences students' preventive practices for internet addiction were affected by an educational intervention (knowledge and health beliefs). Results show that students' health beliefs and knowledge considerably reduce the prevalence of internet addiction. Liu et al. [43] examined the relationship between health literacy and internet addiction among middle school students in Chongqing, China. Findings show that critical health knowledge is negatively correlated with students’ addiction to the internet. Similar findings have been reported by Liu et al. [42].

### Impact of mental health on undergraduates’ Internet addiction

Strong links between internet addiction and mental health problems, such as depression, anxiety, increased stress, and a deterioration in general well-being, have been demonstrated by research findings [4, 41, 69]. Users' conduct and cognitive abilities are disrupted by the psychological condition brought on by internet addiction [74]. The effects of internet addiction on people's mental health, academic performance, interpersonal relationships, and general quality of life have garnered significant attention in the domains of education and mental health in recent years [20, 27, 67].

Kumar et al. [36] examined the relationship between internet addiction and students’ mental health. The findings of the study indicate that addiction to the internet harms mental health and academic performance. The studies by Fantaw [24] and Aderinto [4] also affirm the negative impact of internet addiction on individuals’ mental health. Alavi et al. [7] investigated the relationship between internet addiction disease and psychiatric symptoms in university students in Isfahan. The results of the study indicate a favourable correlation between students' internet addiction and mental health issues. Chen and Zhang [17] investigated the variables affecting college students' internet addiction. The results show that social support, anxiety, pleasure, stress, self-control, and self-efficacy are all important predictors of internet addiction.

### Moderating effect of health knowledge and mental health on the interaction between undergraduates’ attitude to help-seeking and internet addiction

People with mental health issues are urged to seek mental health services for early assessment and intervention to support their psychological well-being, as neglecting to do so can result in more significant treatment gaps and detrimental consequences [54]. The first stage in assessing one's mental health, obtaining a precise diagnosis, and receiving professional assistance for mental health issues is seeking mental health support [50]. Beatie et al. [12] investigated the moderator analysis of the association between young adults' attitudes and behaviours toward obtaining mental health treatment. The findings indicated that mental health knowledge was one of the characteristics that predicted help-seeking attitudes and moderated the association between help-seeking behaviours and attitudes. Yurtseven Yılmaz and Yıldız [77] examined the mediating role of locus of control on the relationship between attitude towards reading and digital addiction in pre-service teachers. The findings indicate that digital addiction is negatively correlated with attitude towards reading, and this relationship is partially mediated by locus of control.

Abd El Salam et al. [1] and Iswanto and Ayubi [30] examined the relationship between mental health literacy and help-seeking behaviour. Findings indicate a positive relationship. Wodong and Utami [72] examined the attitudes toward getting professional help concerning age, gender, education, perceived public stigma, and mental health awareness. Results show that attitudes toward formal help-seeking may be predicted by education, gender, and mental health awareness. Potential predictors of help-seeking behaviour among individuals with mental health issues were investigated by Doll et al. [19]. The study's results showed that functional impairment was the model's best predictor of help-seeking. There was no correlation between help-seeking intentions and perceived or personal stigma.

Guo et al. [29] examined the links between severe internet addiction and negative outcomes related to mental health. Results support a robust link between internet addiction and mental health. Priego-Parra et al. [59] examined the association between internet addiction, anxiety, and depression among the Mexican population during the COVID-19 outbreak. Results indicate a positive relationship. According to research by Sancheti et al. [64], internet addiction is closely linked to anxiety, stress, and sadness. Similar results have been reiterated by Al Mansoor [8], Sharma et al. [65], and Essel et al. [22].

### Theoretical framework

The Uses and Gratification, and the Functional-Interactive-Critical Health Literacy theories are the foundation for this research. According to Blumler and Katz's [13] Uses and Gratification Theory, people utilise social media and the internet for various reasons. Wei et al. [70] assert that the idea eliminates the notion that the media may have an unethical impact on our lives and worldview. This idea holds that people actively select media that meet their unique needs and objectives rather than being passive consumers of information [70]. The notion that people only use the media to fulfill a particular demand appears to undervalue the media's influence in modern society [51]. Both educational and non-educational goals can be pursued by students when they use the media, in this case, the internet. If this is not controlled, they are more likely to develop internet addiction, which will ultimately have a detrimental effect on their mental health.

Kickbusch and Maag [34] introduced the Functional-Interactive-Critical Health Literacy theory. According to the theory, people should be able to make informed decisions about their health using the resources available in their homes, communities, workplaces, schools, and medical facilities. This theory aims to empower people to take charge of their health and determine what actions should be taken to maintain their well-being. According to this study, students' health information is crucial for their decision-making regarding their mental health state. They would also use this knowledge to seek treatment when needed and to exercise self-control over their propensity for internet addiction.

Health knowledge (particularly mental health literacy) and mental health status may moderate the relationship between help-seeking attitudes and internet addiction. This is because mental health literacy improves individuals' ability to identify problematic internet use as a mental health issue requiring intervention [5]. This awareness strengthens the link between positive help-seeking attitudes and reduced addictive behaviours, as informed individuals are more likely to translate their willingness to seek help into concrete actions [75]. Higher mental health literacy decreases stigma associated with seeking professional help, enabling individuals with positive attitudes to overcome social barriers and access treatment [5]. Thus, a moderation framework, as conceived in this study, suggests that improving mental health literacy and addressing comorbid conditions could optimise the effectiveness of help-seeking attitudes in combating internet addiction.

### Hypotheses

- H1:Attitude to help-seeking will significantly impact undergraduates’ internet addiction.
- H_2_: Health knowledge will significantly impact undergraduates’ internet addiction.
- H_3_: Mental health will significantly impact undergraduates’ internet addiction.
- H_4_: Health knowledge will significantly moderate the interaction between undergraduates’ attitudes toward help-seeking and internet addiction.
- H_5_: Mental health will significantly moderate the interaction between undergraduates’ attitudes to help-seeking and internet addiction.

## Methodology

### Research design

This study used a correlational research design, which is non-experimental.

### Population, sampling techniques, and sample

The study population consisted of undergraduates from public federal universities in South-West Nigeria. From the existing cluster of states in the South-West, Nigeria, that is: Ondo/Ekiti, Lagos/Ogun, and Oyo/Osun), purposive sampling technique was used to select three (3) states (Ekiti, Lagos, and Oyo) which comprise one public federal university each that does not share geographical communality. A Google Form link was randomly disseminated to undergraduates’ Telegram and WhatsApp platforms through their lecturers and Faculty Officers in the selected universities. The inclusion criteria for the study were undergraduate students in public universities who must have an internet-enabled phone. The exclusion criteria consist of postgraduate students, students in private universities or other tertiary institutions, and those without internet-enabled phones. Through a convenience sampling, a total of 1,684 undergraduates participated in the study.

### Instruments

The Health Knowledge Questionnaire (HKQ), Mental Health Questionnaire (MHQ), the Internet Addiction Questionnaire (IAQ), and Attitude towards Help-Seeking Questionnaire (AHSQ) were the tools utilised to gather data.

#### Health Knowledge Questionnaire (HKQ)

The HKQ was adapted from the published article of Onwe and Okocha [55], which had nine items on a 5-Likert scale of Very Low, Low, Moderate, High, and Very High. With fourteen (14) items on a 4-Likert scale format—Bottom 10% (scoring as 1), Below Average (scored as 2), Above Average (scored as 3), and Top 10% (scores as 4)—the HKQ was adapted for this study to assess undergraduates' health knowledge level. A higher score indicates high or very high health knowledge.

#### Mental Health Questionnaire (MHQ)

The MHQ was adapted from the published work of Lukat et al*.* [44], which had nine items on a 4-Likert scale of Very True of Me, True of Me, Rarely True of Me, and Not True of Me. With thirteen (13) items on a 4-Likert scale format—Not True of Me (scoring as 1), Rarely True of Me (scored as 2), True of Me (scored as 3), and Very True of Me (scores as 4)—the MHQ was altered for this study to assess the mental health state of undergraduates.

### Attitude towards Help-Seeking Questionnaire (AHSQ)

The AHSQ was adapted from the published work of Wilson et al*. *[71]*,* which had twelve items on a 5-Likert scale of Very Unlikely to Very Likely. A higher score indicates high mental health. Using sixteen (16) items on a 4-Likert scale format of Very Unlikely (scoring as 1), Unlikely (scored as 2), Likely (scored as 3), and Very Likely (scores as 4), the AHSQ was tweaked for this study to gauge undergraduates' attitudes towards obtaining care for their medical concerns. High score indicate favourable attitudes toward help-seeking.

### The Internet Addiction Questionnaire (IAQ)

The IAQ is an adapted instrument that Pee and Shafeq modified from the original version credited to Young (1998). It was designed to measure students’ internet addiction. Sixteen questions about student internet addiction make up the test, with response format of Strongly Agree (rated as 4), Agree (scoring as 3), Disagree (ranked as 2), and Strongly Disagree (scored as 1). Students were considered to have an online addiction for this study if they met these two inclusion criteria: an IA score of > 65 and daily internet usage of 5 h. A high score indicates excessive addiction to the internet.

In one of the sampled universities, five specialists in item development and Tests, Measurement, and Evaluation in the Faculties of education (3) and psychology (2) determined the face and content validities of the instruments. Although previous studies have standardised these instruments, for this study’s context, we pilot tested them on a small sample of 63 undergraduates at a university in the middle belt of Nigeria (outside this current study’s site to avoid sample contamination). Furthermore, because the instruments are ordinal, their internal consistency was determined using R software's Ordinal Alpha reliability estimates, yielding Health Knowledge (α = 0.94), Mental Health (α = 0.91), Attitude to Help-Seeking (α = 0.84), and Internet Addiction (α = 0.82), respectively.

### Procedure for data collection

The office of the dean of the Faculty of Education at the sampled universities provided written approval and ethical clearance for this study (FUOYE/ED/2024/06/0017). The instruments were created using Google Forms, an internet tool. Informed consent was obtained from the sampled undergraduates, the purpose of the study was made known, and voluntary participation was requested through the various undergraduates’ communication group platforms (Telegram, WhatsApp, and Google Classrooms). Additionally, a voluntary participation option was added to the Google link form, allowing users to choose whether or not to accept participation and continue with the online survey. After lectures, course instructors reminded students to participate to increase the response rate. The three-month data collection period ran from November 2024 to January 2025.

### Method of data analysis

Because of its unique method for handling continuous, ordinal, and categorical data to establish interaction/moderation effects, the Hayes macro process in SPSS version 26.0 was used to analyse the data. The characteristics of the respondents were summarised using descriptive statistics, and hypotheses were answered at a 0.05% significance level using inferential statistics (Hayes macro process V4.0, Model 2).

### Conceptual and statistical diagrams

**Fig. 1:**
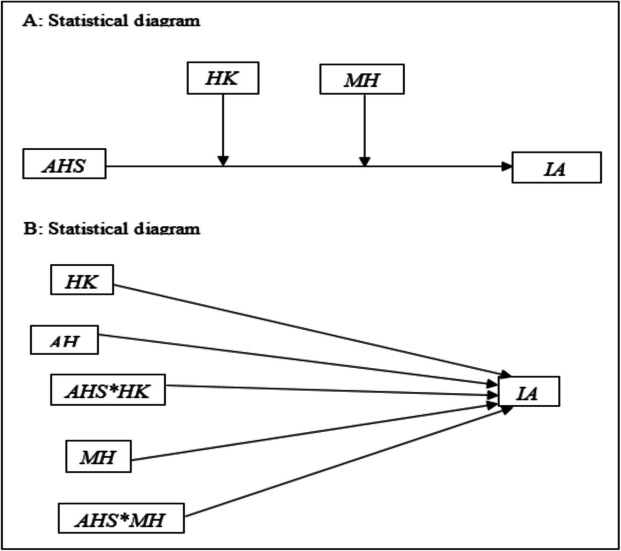
Conceptual and statistical diagrams. Sources: Researchers conceptualisation (2025). Where: AHS: Attitude to Help-Seeking (Independent), HK: Health Knowledge (Moderator 1), MH: Mental Health (Moderator 2), IA: Internet Addiction (Dependent)

Figure 1 depicts the conceptualisation of the variables in this study. The figure examines the direct impact of attitude towards help-seeking, health knowledge, and mental health on internet addiction. Also, the interaction/moderating effect of health knowledge (HK) and mental health (MH) on the association between attitude towards help-seeking (AHS) and internet addiction (IA).

## Results

Indicated in Table 1 is the demographic information of this study’s respondents. Accordingly, concerning gender, 772 (45.84) of the respondents were males while 912 (54.16) were females. This implies that more female respondents participated in this study. Regarding the university of the respondents, 764 (45.35) were from the Federal University Oye-Ekiti, in Ekiti State; 234 (13.90) were from the University of Lagos, Lagos State, while 686 (40.74) of the respondents were from the University of Ibadan, Oyo State. As such, most of the study’s respondents were from the Federal University Oye-Ekiti, in Ekiti State.

**Table 1:** Respondents’ demographic information

Variable	Status	Frequency	Percentage
Gender	Male	772	45.84
	Female	912	54.16
University	Federal University Oye-Ekiti	764	45.37
	University of Lagos	234	13.90
	University of Ibadan	686	40.74

### Hypotheses

- H_1_:Attitude towards help-seeking will significantly impact undergraduates’ internet addiction.

Presented in Table 2 are the results on the impacts of attitude towards help-seeking, health knowledge, and mental health on internet addiction among undergraduates in the sampled universities. Results in the Table reveal that the overall relationship was weak (*R* = 0.07), and not significant (*F* = 1.74; *p* > 0.05). Furthermore, the result of H_1_ in the Table reveals that attitude towards help-seeking significantly impacted internet addiction (b = 0.04, t = 2.14, *p* < 0.05), though low. Hence, this hypothesis was supported. This implies that the attitude towards help-seeking impacted undergraduates' addiction to the internet.

**Table 2:** Impact of direct predictor variables on internet addiction

**Model Summary**						
**R** 0.07	**R-sq** 0.01	**MSE** 30.33	**F** 1.74	**Df1** 5.00	**Df2** 1678.00	**P** 0.12
	**Coeff**	**Se**	**T**	**P**	**LLCI**	**ULCI**
Constant	39.05	0.14	279.72	0.00	38.78	39.33
Attitude to help-seeking	0.04	0.02	2.14	0.03	0.00	0.07
Health knowledge	-0.01	0.01	-0.46	0.64	-0.03	0.02
Mental health	0.00	0.03	-0.15	0.88	-0.05	0.05

- H_2_: Health knowledge will significantly impact undergraduates’ internet addiction.

The result of H_2_ in Table 2 shows that health knowledge had a negative and insignificant impact on internet addiction (b = -0.01, t = -0.46, *p* > 0.05). Hence, this hypothesis was rejected. This implies that the health knowledge of undergraduates did not impact their addiction to the internet.

- H_3_: Mental health will significantly impact undergraduates’ internet addiction.

The result of H_3_ in Table 2 reveals that mental health had a positive but insignificant impact on internet addiction (b = 0.00, t = -0.15, *p* > 0.05). Hence, this hypothesis was rejected. This implies that mental health did not impact internet addiction among undergraduates in the sampled public universities.

- H_4_: Health knowledge will significantly moderate the interaction between undergraduates’ attitudes toward help-seeking and internet addiction.

Presented in Table 3 are the results on the interaction of health knowledge of undergraduates (moderator 1) on the relationship between attitude to help-seeking and internet addiction. The variables were centered to avoid potential multicollinearity with the interaction term (Aiken & West, 1991). Therefore, the result of H_4_ reveals a positive but insignificant moderating impact of health knowledge on the relationship between attitude towards help-seeking and internet addiction (b = 0.00, t = 1.00, *p* > 0.05). Similarly, the interaction term accounted for a 0% change in internet addiction (R^2^ = 0.00; *p* > 0.05). Hence, this hypothesis was rejected. This implies that the interaction/moderating impact of attitude towards help-seeking and health knowledge did not account for a significant variance in internet addiction among the sampled undergraduates.

**Table 3:** Interaction of attitude to help-seeking and health knowledge on Internet addiction

	**Coeff**	**Se**	**T**	**P**	**LLCI**	**ULCI**
Constant	39.05	0.14	279.72	0.00	38.78	39.33
Int_1	0.00	0.00	1.00	0.32	0.00	0.00
Test(s) of highest order unconditional interaction(s)						
	R2-chng	F	df1	df2	P	
Attitude to help-seeking*Health knowledge	0.00	1.01	1.00	1678.00	0.32	

*Int_1* Focal Predictor*Moderator 1

- H_5_: Mental health will significantly moderate the interaction between undergraduates’ attitudes toward help-seeking and internet addiction.

Indicated in Table 4 are the results of the interaction of mental health on the relationship between attitude to help-seeking and internet addiction among undergraduates in the sampled universities. Therefore, the result of H_5_ reveals a negative and insignificant moderating impact of mental health on the relationship between attitude towards help-seeking and internet addiction (b = -0.01, t = -1.63, *p* > 0.05). Also, the interaction term accounted for a 0% change in internet addiction (R2 = 0.00; *p* > 0.05). Hence, this hypothesis was rejected. This implies that the interaction/moderating impact of attitude towards help-seeking and mental health did not account for significant variance in internet addiction among undergraduates as sampled.

**Table 4:** Interaction of attitude to help-seeking and mental health on internet addiction

	**Coeff**	**Se**	**T**	**P**	**LLCI**	**ULCI**
Constant	39.05	0.14	279.72	0.00	38.78	39.33
Int_2	-0.01	0.00	-1.63	0.10	-0.01	0.00
Test(s) of highest order unconditional interaction(s)						
	R2-chng	F	df1	df2	P	
Attitude to help-seeking* Mental health	0.00	2.67	1.00	1678.00	0.10	

*Int_2* Focal Predictor*Moderator 2

## Discussion

Internet addiction is a global challenge, especially among university students [17, 49, 63]. This study has examined the moderating role of mental health and health knowledge on the relationship between undergraduates' attitudes toward help-seeking and internet addiction in selected public universities in southwest Nigeria. A finding of the study indicates that undergraduates’ attitudes toward help-seeking impacted their addiction to the internet. Since a problem shared is half solved, students who are already addicted to the internet can be helped if they possess a positive attitude towards help-seeking. Thus, internet addiction levels of undergraduates were significantly influenced by their attitudes toward help-seeking. This finding is consistent with those of Chen and Zhang [17], Lu et al. [45], and Özer et al. [56], which indicated a positive predictive relationship between social support available to students and internet addiction. Undergraduates’ reluctance or negative attitudes toward help-seeking for psychological or behavioral issues directly contribute to higher levels of internet addiction. This is due to a combination of emotional distress, lack of social support, and reliance on the internet as a maladaptive coping strategy. Addressing these attitudes and promoting help-seeking behaviours are essential for reducing internet addiction and improving student well-being [5, 53].

Another finding of this study reveals that health knowledge did not impact undergraduates’ internet addiction. One's knowledge about health does not have a connection with behavioural disorders such as internet addiction. Students will not fully comprehend the risks and consequences of internet addiction despite having general health knowledge. Students might believe that internet addiction is not a serious health issue or that it does not apply to them. The pleasure students derive from being addicted to the internet is not controlled by the knowledge of health they have, even against such addictive behaviour. This finding contrasts with those of Langarizadeh et al. [40], Liu et al. [42, 43], who reported a negative correlation between internet addiction and students’ health knowledge or literacy. This difference in findings might be because students' susceptibility to internet addiction can vary greatly due to personality differences, coping mechanisms, environment, social factors, and life experiences.

A finding of this study reveals that mental health also did not impact internet addiction among undergraduates. Undergraduate addiction to the internet can become a deeply ingrained habit, making it difficult to change such behaviour even with stable mental health. They might not recognize their internet use as problematic or addictive, irrespective of their mental health status. Many students use the internet to cope with stress, boredom, social isolation, or other emotions. Especially in the context of this study, the internet provides a sense of social connection and community for these students, which can be beneficial for their mental health, but does not necessarily impact internet addiction. This finding contradicts those of Alavi et al. [7] and Chen and Zhang [17], which indicated a positive relationship between student mental health problems and their addiction to the internet. This current study’s concern was mainly on mental health (and not mental health problems), which could explain the difference in the findings.

A finding of this study also depicts that the interaction/moderating impact of attitude towards help-seeking and health knowledge did not account for a significant variance in internet addiction among the sampled undergraduates. Research identifies psychosocial variables, such as stress, self-control, pleasure-seeking, anxiety, self-efficacy, depression, social anxiety, loneliness, poor social support, dissatisfaction with academic major, and the purposes for which the internet is used (e.g., entertainment, social networking)-as more significant predictors of internet addiction [9, 35, 47]. While help-seeking attitudes and health knowledge are important for general well-being, they may not directly address or mitigate excessive internet use's psychological and behavioural drivers. For example, students may be aware of the risks of internet addiction and have a positive attitude toward seeking help. However, if they are experiencing high stress, loneliness, or poor self-control, these factors can override their intentions or knowledge, leading to problematic internet use [9, 47]1. Additionally, the accessibility of the internet, peer influences, and specific online activities (such as gaming or social media use) have been shown to play a more immediate role in fostering addictive behaviours than general health knowledge or attitudes toward seeking help [37].

However, a positive but insignificant association was also revealed. These students may not have recognized their internet use as problematic or addictive, making their help-seeking attitudes less relevant. Fear of being judged or labeled as "internet-addicted" might prevent them from seeking help, regardless of their attitude towards help-seeking, as they may believe they can overcome internet addiction on their own, reducing the need to seek help. Health knowledge does not necessarily translate into behaviour change or action, and these students may have prioritised other aspects of their lives over their health, reducing the influence of health knowledge on internet addiction. This finding is in tandem with those of Abd El Salam et al. [1], Doll et al. [19], Essel et al. [22], Guo et al. [29], Iswanto and Ayubi [30], Mansoor (2023), Priego-Parra et al. [59], Sancheti et al. [64], Sharma et al. [65], and Wodong and Utami [72], which revealed a positive association between mental health literacy and help-seeking behaviour. The finding that the interaction/moderating impact of attitude towards help-seeking and mental health did not account for significant variance in internet addiction among undergraduates contradicts the findings of Beatie et al. [12] and Yurtseven Yılmaz and Yıldız [77] that revealed that mental health knowledge was one of the characteristics that predicted help-seeking attitudes and that it also moderated the association between help-seeking behaviours.

### The study’s implications

According to the study's findings, a concentrated effort is required to enhance undergraduate mental health support, including well-informed tactics to encourage positive attitudes toward help-seeking and lessen the stigma associated with mental health conditions. By emphasising the value of health knowledge in preventing internet addiction and fostering undergraduates' mental well-being, universities may further advance/improve their health education programmes. To encourage undergraduates to use the internet healthily, university psychologists and medical professionals should determine possible risk factors and preventative measures and make these known to students. Support for public health campaigns and educational initiatives that aim to improve mental health and healthy internet use habits, as well as legislation that restricts internet use, healthy internet habits, and avoidance of internet addiction, should be encouraged in universities. Universities should also provide diagnostic criteria and mental health evaluation tools for internet addiction, as well as efficient therapies and interventions for the condition, such as family-based interventions and cognitive-behavioural therapies.

### Limitations of the study

This study has some limitations, which must be considered while deliberating on the findings or their transferability. Respondents’ self-reported data may be subject to biases (e.g., social desirability or recall bias), the sample may not be representative of the larger population, limiting generalisability, and the study's cross-sectional design may not capture the dynamic relationships between the studied variables over time. Also, the study may not have captured the long-term effects of mental health, health knowledge, attitudes toward help-seeking, and internet addiction. The use of only on-campus data collection may have also impacted this study’s results. Future research should improve upon these.

## Conclusion and recommendations

This study has investigated the moderating role of mental health and health knowledge on the relationship between undergraduates' attitudes toward help-seeking and internet addiction in three public universities in southwest Nigeria. Given the scanty evidence on the subject, the findings of this study have revealed that undergraduates’ attitudes toward help-seeking impact their addiction to the internet, health knowledge does not impact undergraduates’ internet addiction, mental health does not impact internet addiction among undergraduates, and health knowledge and mental health of undergraduates have no moderation effect on the relationship between attitude towards help-seeking and internet addiction. Based on these findings, we recommend that further awareness campaigns and programmes that enlighten students about the adverse effects of internet addiction, as well as those that boost their positive attitudes toward help-seeking, be organised and sustained in Nigerian public universities. An enabling environment that fosters undergraduates’ positive attitudes toward help-seeking should be provided. Incentives should be given to students who seek help regarding their addictions to the internet. Universities’ health/counseling/help centers should provide mechanisms that boost more positive help-seeking attitudes among students. Digital well-being programmes should be orsganised in Nigerian universities to address internet addiction tendencies. Counseling services specifically for internet addiction cases, and university-led mentorship programmes that encourage help-seeking attitudes among undergraduates, should be advanced.

## Supplementary Information

Supplementary Material 1.

## Data Availability

Data for this research will be made available upon reasonable request from the corresponding author.
