# Supplementary material for: Moderating effect of health knowledge and mental health on the association between undergraduates’ attitudes toward help-seeking and internet addiction
Source: BMC Public Health. 2025 Jul 2;25:2290. doi: 10.1186/s12889-025-23538-x (PMC12219965; doi:10.1186/s12889-025-23538-x)
Supplement: Supplementary file 1 — Supplementary Material 1. [file 12889_2025_23538_MOESM1_ESM.docx]

**Health Knowledge Questionnaire (HKQ)**

*Dear Respondents,*

*This questionnaire elicits responses on Health Knowledge among undergraduates in Nigerian public universities in Ekiti State. You are expected to respond to all the items on the questionnaire by picking the appropriate option. Your response will be treated with utmost confidentiality and used for research purposes only.*

*Thank you.*

**Health Knowledge**

| **S/No** | **Items**  *What is the level of your health knowledge compared to average person in your school about* | **In the top 10%** | **Above Average** | **Below Average** | **In the bottom 10%** |
| --- | --- | --- | --- | --- | --- |
| 1 | Sexual health |  |  |  |  |
| 2 | Reproductive health |  |  |  |  |
| 3 | Oral health |  |  |  |  |
| 4 | Dental health |  |  |  |  |
| 5 | Preventive health |  |  |  |  |
| 6 | Nutrition health |  |  |  |  |
| 7 | Exercises for healthy body system |  |  |  |  |
| 8 | Stress management |  |  |  |  |
| 9 | Emotional well-being |  |  |  |  |
| 10 | Mental health |  |  |  |  |
| 11 | Tobacco consumption |  |  |  |  |
| 12 | Alcohol consumption |  |  |  |  |
| 13 | Drug use |  |  |  |  |
| 14 | General health |  |  |  |  |

**Mental Health Questionnaire (MHQ)**

*Dear Respondents,*

*This questionnaire is designed to elicit responses on Mental Health among undergraduates in Nigerian public universities in Ekiti State. Please, you are expected to respond to all the items on the questionnaire by picking the appropriate option. Your response will be treated with utmost confidentiality and used for research purpose only.*

*Thank you.*

**Mental Health**

| **S/N** | **Items**  *When it comes to my mental health, I am* | **Very True of Me** | **True of Me** | **Rarely True of Me** | **Not True of Me** |
| --- | --- | --- | --- | --- | --- |
| 1 | Carefree |  |  |  |  |
| 2 | Mindful of been in good life |  |  |  |  |
| 3 | Eager to enjoy my life |  |  |  |  |
| 4 | Satisfied with my being |  |  |  |  |
| 5 | Confident of healthy living |  |  |  |  |
| 6 | Meticulous to source for my needs |  |  |  |  |
| 7 | In good physical condition |  |  |  |  |
| 8 | In good emotional condition |  |  |  |  |
| 9 | In good psychological condition |  |  |  |  |
| 10 | Well equipped to deal with |  |  |  |  |
| 11 | Willing to do whatever brings me joy |  |  |  |  |
| 12 | A calm human being |  |  |  |  |
| 13 | A balanced human being. |  |  |  |  |

**Attitude to Help-Seeking Questionnaire (AHSQ)**

*Dear Respondents,*

*This questionnaire is designed to elicit responses on the disposition towards Help-Seeking about health conditions among undergraduates in Nigerian public universities in Ekiti State. You are expected to respond to all the items on the questionnaire by picking the appropriate option. Your response will be treated with utmost confidentiality and used for research purposes only.*

*Thank you.*

**Attitude towards Help-Seeking Questionnaire (AHSQ)**

| **S/N** | **Items**  *How likely would you seek help about your health conditions from the following?* | **Very Likely** | **Likely** | **Unlikely** | **Very Unlikely** |
| --- | --- | --- | --- | --- | --- |
| 1 | Intimate partner |  |  |  |  |
| 2 | Friends in school |  |  |  |  |
| 3 | Friends at home |  |  |  |  |
| 4 | Health care professionals |  |  |  |  |
| 5 | Health care centre in the school |  |  |  |  |
| 6 | Health care centre outside the school |  |  |  |  |
| 7 | Religious leaders |  |  |  |  |
| 8 | Parents |  |  |  |  |
| 9 | Family relations |  |  |  |  |
| 10 | Phone/helpline on health related services |  |  |  |  |
| 11 | Medical doctors |  |  |  |  |
| 12 | Nurses |  |  |  |  |
| 13 | A Psychologist |  |  |  |  |
| 14 | A Counsellor |  |  |  |  |
| 15 | I prefer to seek help from others not listed above |  |  |  |  |
| 16 | I prefer not to seek help from anyone |  |  |  |  |

**Internet Addiction Questionnaire (IAQ)**

*Dear Respondents,*

*This questionnaire is designed to elicit responses on Internet Addiction among undergraduates in Nigeria. You are expected to respond to all the items on the questionnaire by picking the appropriate option. Your response will be treated with utmost confidentiality and used for research purposes only. Thank you.*

**Internet Addiction**

| **S/N** | **Items** | **Strongly Agree** | **Agree** | **Disagree** | **Strongly Disagree** |
| --- | --- | --- | --- | --- | --- |
| 1 | I spend long hours daily on the internet. |  |  |  |  |
| 2 | The Internet always makes me lonely. |  |  |  |  |
| 3 | All my school work is done using the internet |  |  |  |  |
| 4 | I find it easier to get academic information from the internet. |  |  |  |  |
| 5 | I watch YouTube daily. |  |  |  |  |
| 6 | I hardly ask for food while on the internet. |  |  |  |  |
| 7 | I have many friends on the social network. |  |  |  |  |
| 8 | I stay on the internet till daybreak. |  |  |  |  |
| 9 | I have no friends on social media. |  |  |  |  |
| 10 | I used many data daily to browse |  |  |  |  |
| 11 | I prefer to be absent from class most of the time because I can get better information from the Internet. |  |  |  |  |
| 12 | I have three Handsets used to browse. |  |  |  |  |
| 13 | I stay back in the town because of the poor/no network in my locality |  |  |  |  |
| 14 | I spent much maintaining my phone to avoid being cut off from the internet. |  |  |  |  |
| 15 | I watch and chat on Facebook all-day |  |  |  |  |
| 16 | I used internet service every day of the week |  |  |  |  |
